# Point-of-care cervical cancer screening using deep learning-based microholography

**DOI:** 10.7150/thno.37187

**Published:** 2019-11-26

**Authors:** Divya Pathania, Christian Landeros, Lucas Rohrer, Victoria D'Agostino, Seonki Hong, Ismail Degani, Maria Avila-Wallace, Misha Pivovarov, Thomas Randall, Ralph Weissleder, Hakho Lee, Hyungsoon Im, Cesar M. Castro

**Affiliations:** 1 Center for Systems Biology, Massachusetts General Hospital, Boston, MA 02114, USA; 2 Harvard-MIT Program in Health Sciences and Technology, Massachusetts Institute of Technology, Cambridge, MA 02139, USA; 3 Department of Health Sciences, Northeastern University, Boston, MA 02115, USA; 4 Department of Bioengineering, Northeastern University, Boston, MA 02115, USA; 5 Department of Electrical Engineering and Computer Science, Massachusetts Institute of Technology, Cambridge, MA 02139, USA; 6 Department of Obstetrics and Gynecology, Massachusetts General Hospital, Boston, MA 02114, USA; 7 Department of Systems Biology, Harvard Medical School, 200 Longwood Ave, Boston, MA 02115, USA; 8 Department of Radiology, Massachusetts General Hospital, Boston, MA 02114, USA; 9 Massachusetts General Hospital Cancer Center, Harvard Medical School, Boston, MA 02114, USA

**Keywords:** Cervical cancer, point-of-care screening, global oncology, microholography, deep learning

## Abstract

Most deaths (80%) from cervical cancer occur in regions lacking adequate screening infrastructures or ready access to them. In contrast, most developed countries now embrace human papillomavirus (HPV) analyses as standalone screening; this transition threatens to further widen the resource gap.

**Methods:** We describe the development of a DNA-focused digital microholography platform for point-of-care HPV screening, with automated readouts driven by customized deep-learning algorithms. In the presence of high-risk HPV 16 or 18 DNA, microbeads were designed to bind the DNA targets and form microbead dimers. The resulting holographic signature of the microbeads was recorded and analyzed.

**Results:** The HPV DNA assay showed excellent sensitivity (down to a single cell) and specificity (100% concordance) in detecting HPV 16 and 18 DNA from cell lines. Our deep learning approach was 120-folder faster than the traditional reconstruction method and completed the analysis in < 2 min using a single CPU. In a blinded clinical study using patient cervical brushings, we successfully benchmarked our platform's performance to an FDA-approved HPV assay.

**Conclusions:** Reliable and decentralized HPV testing will facilitate cataloguing the high-risk HPV landscape in underserved populations, revealing HPV coverage gaps in existing vaccination strategies and informing future iterations.

## Introduction

Prompt and reliable triaging of high-risk HPV cases could help offset severe pathology bottlenecks in resource-limited regions 1,2, and circumvent geographical and/or socioeconomic barriers 3 to effective cervical cancer screening. Visual inspection with acetic acid (VIA) is adopted as a rapid, inexpensive alternative to standard cytology (Pap smears); however, it suffers from high rates of false-negative and false-positive results 4,5. In contrast, DNA testing for high-risk HPV confers superior sensitivity (96-100%) and specificity (90-100%) 6 along with greater clinical benefit when compared to cytology or VIA 7. The HPV-focused work presented in this manuscript is very timely and poised to address clinical diagnostic disparities. The time required for a pre-cancerous cervical lesion to transform into frank cervical cancer reflects a lengthy process. This underscores why every three-year recommended interval for Pap smear testing remains effective despite its lower performance. An important assumption is that women would diligently be screened at such regular intervals; the inherent resource and geopolitical challenges in less developed regions of the world often demand prompt intervention at any given opportunity. Even the guidelines from most developed countries now favor HPV screening instead of Pap smears 8. In 2018, the US Preventive Services Task Force reported that women ages 30 to 65 could be screened for cervical cancer with HPV testing every five years, *without undergoing a simultaneous Pap test* 9. The Lancet Oncology recently published modeling work that concluded that women with negative HPV testing after age 55 had low cervical cancer risk for the remainder of their lives 10. In contrast, women undergoing Pap smear-only screening would still benefit from screening until age 75. As such, accurate and reliable access to HPV testing for the right patients could relieve resource constraints (through many fewer years of screening) domestically and abroad. Yet, HPV testing demands significant equipment and maintenance costs compared to cytology or VIA and is often limited to central laboratories. As such, current “state-of-the-art” cervical cancer screening recommendations are poised to further the divide between resource rich and poor regions.

Recently, digital microholography has been leveraged for high-throughput imaging and sensing applications 11-16. The technology is promising for point-of-care (POC) devices due to its simple optical configuration (*e.g.*, lens and filter free), low cost, wide field-of-view over cm^2^, and high resolution on a sub-micron scale 17. We previously developed a portable molecular diagnostic approach to detect cancer-related surface *protein* markers within clinical *biopsies* using antibodies linked to microbeads 13,18. Target cells were immunolabeled by microbeads, and their hologram (or diffraction) patterns were wirelessly transmitted and digitally reconstructed with “cloud computing” to identify target cells and quantify protein marker expressions.

Here, we describe an approach coined Artificial Intelligence Monitoring for HPV (AIM-HPV), that integrates low and high-tech solutions for DNA-based and POC cancer screening. First, cervical biopsies were replaced by cervical brushings to (i) improve clinical workflow, (ii) maximize patient acceptability/safety, (iii) obviate the need for skilled operators, and (iv) transition from triage to screening. We devised a disposable DNA extraction kit based on manual syringe operations. We developed a new POC microholography platform and assay scheme to detect target nucleic acids. In the presence of HPV 16 and/or 18, microbeads were designed to bind the DNA target and form microbead dimers. The resulting holographic signature was recorded and analyzed. To achieve scalable and low-cost DNA-based cancer screening of populations in areas of greatest need, we leveraged machine learning strategies to enable complete on-site analytics. This eliminated the need for cloud computing and attendant high fixed-costs, thus rendering a genuine POC screening strategy unencumbered by wireless data services or expensive computational resources (*e.g.*, GPU servers). Through a proof-of-concept clinical study using cervical brushings from high-risk patients, we successfully benchmarked our platform's performance to an FDA-approved HPV screening test.

## Results

### AIM-HPV device and method for HPV DNA detection

**Figure 1:**
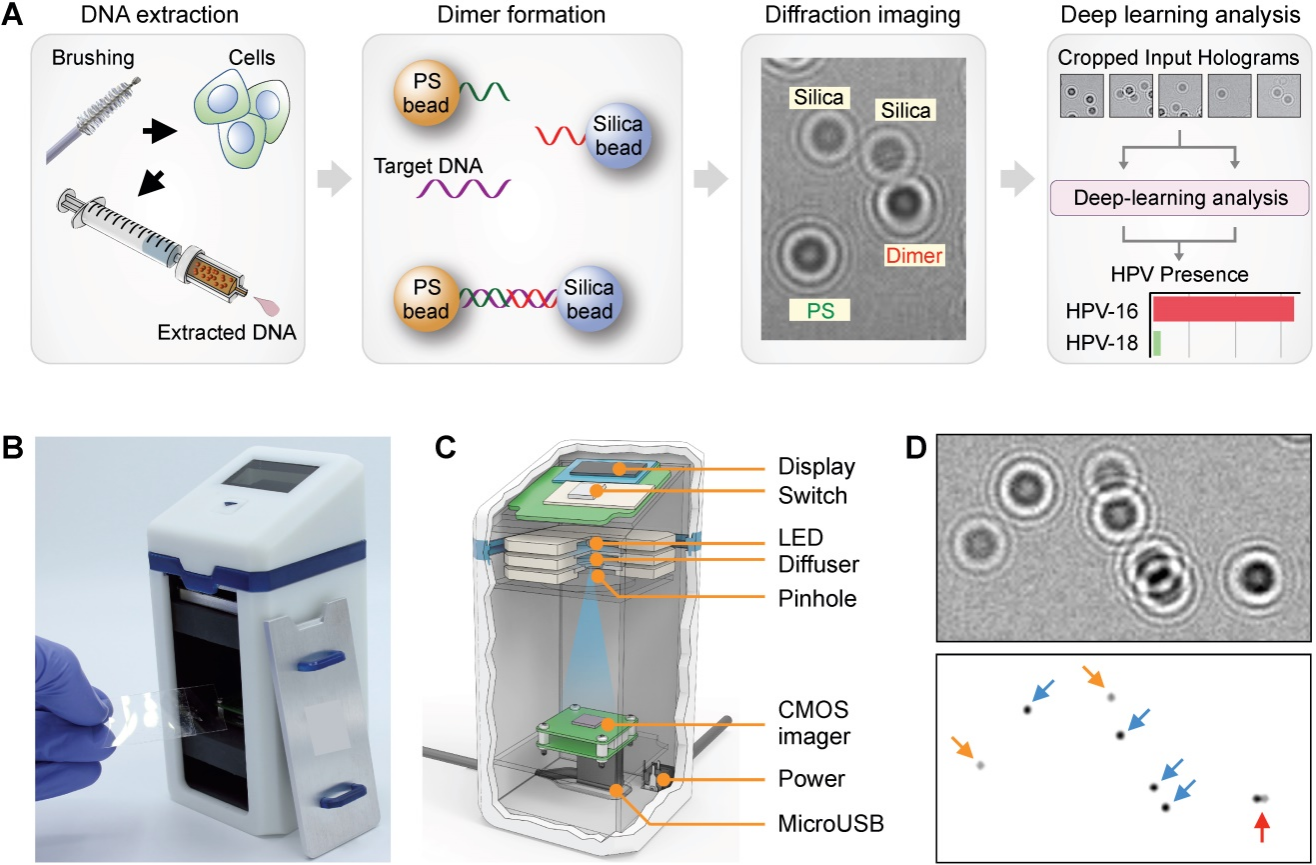
** Artificial intelligence monitoring for human papillomavirus (AIM-HPV) assay. (A)** Assay procedure. From cells obtained by cervical brushing, DNAs were extracted using a disposable bead-filter device. In the presence of target DNA (HPV16 and 18), PS and silica beads coated with DNA probes complementary to the 3' and 5' ends of the HPV target DNA formed dimers. Diffraction patterns of PS, silica, and PS-silica dimers were recorded and analyzed by deep-learning algorithms. **(B, C)** The AIM-HPV device in photograph **(B)** and schematic **(C)** is equipped with a light source (LED, diffuser, pinhole) and image sensor for recording diffraction patterns of beads. **(D)** Diffraction patterns of PS beads (blue arrow), silica beads (orange), and PS-silica bead dimer (red) and their corresponding microscopic image.

The overall workflow of our AIM-HPV method with its advanced features is depicted in Figure 1A. First, during a pap-smear procedure, cervix cells were collected by a cervical brush, and their DNAs were extracted using a disposable syringe-filter. After asymmetric DNA amplification, DNA samples were mixed with 6 µm polystyrene (PS) and 5 µm silica beads, each coated with DNA probes complementary to the 3' and 5' ends of the target HPV DNA, respectively. In the presence of target DNA, the two types of beads are linked through the target DNA and form detectable PS-silica beads dimers. Diffraction patterns of PS, silica, and PS-silica bead dimers were captured by our a miniaturized microholography device and quickly analyzed by trained deep-learning algorithms.

The DNA extraction device is built on a bead-loaded disposable filter to directly isolate DNA from cell lysates (Figure S1) 19. We used poly(methyl methacrylate) microbeads (PMMA, 14.7 µm in diameter) coated with a silica layer via polydopamine to capture negatively charged DNA on the silica surface. The absorbed DNA was then eluted by changing the salt concentration in water. The total amount of DNA extracted from cells (200 µl, 5 × 10^6^ cells) using the bead filter was 137.7 ng/µl, comparable with the amount extracted using a commercial Qiagen kit (179.1 ng/µl). In terms of the final assay signals, we saw no significant difference (unpaired t-test, *p* = 0.4582) between HPV DNA isolated by either method.

We custom-designed our AIM-HPV device with a focus on potential applications in resource-limited settings (Figure 1B). Compared to a previous protein-focused holographic device^13^, the new platform offers better optics (*e.g.*, high-power LED, CMOS image sensor with more pixels) controlled by a one-touch switch and can readily link to tablets or laptop computers (Figure 1C). The overall dimensions of the device were 65 mm (L) × 65 mm (W) × 140 mm (H) and 0.6 kg in weight, thus allowing for portable use. With the AIM-HPV device, hologram patterns of PS beads, silica, and their dimers were recorded for subsequent deep learning analysis (Figure 1D). HPV 16 and 18 levels (*S*) were estimated by dividing the number of dimers (*di*) with the product of PS (*ps*) and silica (*si*) bead counts: *S* = *k*·*di*·(*ps*·*si*)^-1^, where *k* is a constant value of 10^5^ to scale it to 0-100. We used a housekeeping gene (*i.e.*, β-globin) as a positive control for the assay. Without any DNA, the level of HPV signal was about 0.8 ± 0.5 (mean ± s.d.; Figure S2).

### Rapid analysis by customized deep-learning algorithms

**Figure 2:**
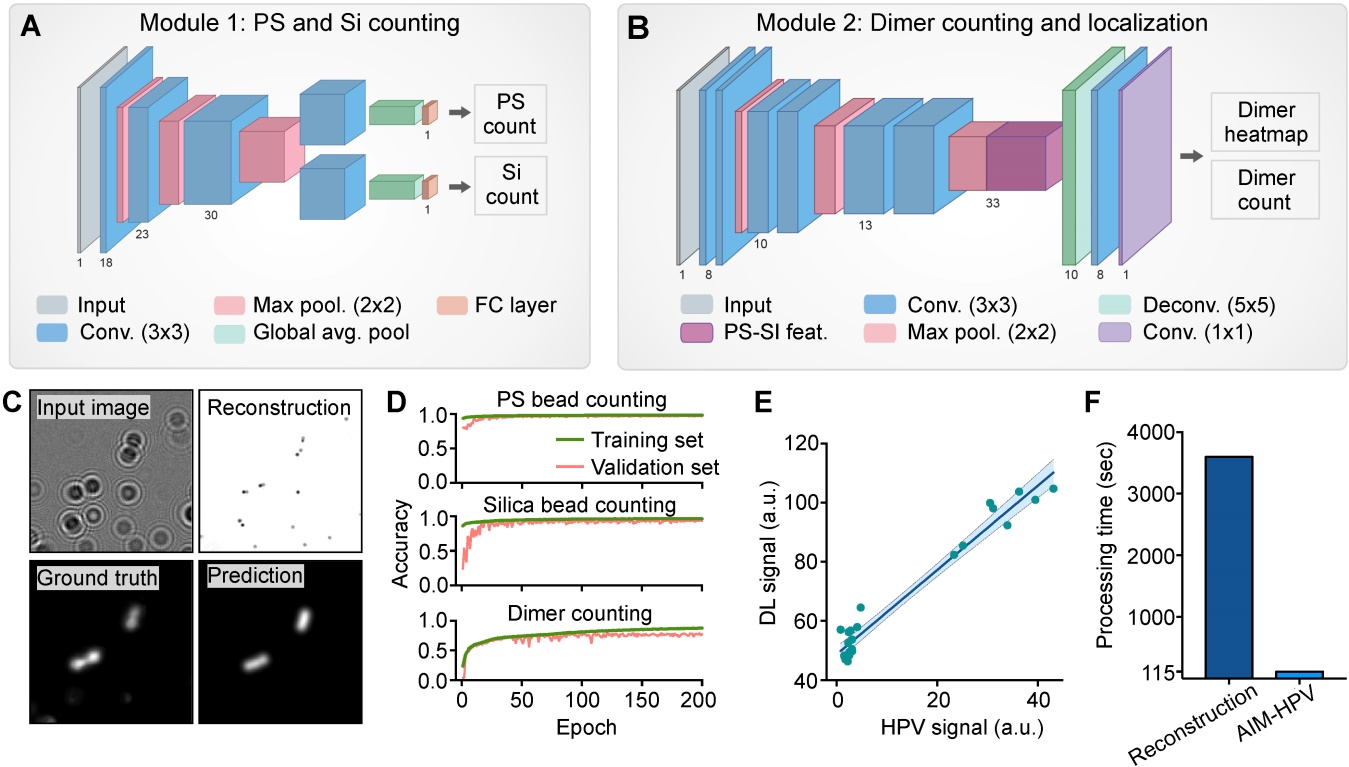
** Convolutional Neural Network for Clinical Sample Analysis. (A, B)** Schematics outline models for PS/Si counting individually **(A)** and PS-Si dimer localization and counting **(B)**. The latter produces a heat map of dimer locations in which the sum of all pixels is taken as the total dimer count. **(C)** The clinical sample image on the left is reconstructed and shown on the right. The ground truth heat map (generated from reconstruction coordinates) and model-predicted heat map are shown. **(D)** Accuracy of PS, silica, and dimer bead detection over training epochs. **(E)** The final AIM-HPV signal values, calculated from convolutional network counts, are plotted against the HPV signal calculated from the reconstruction method. **(F)** Computation time to generate final AIM-HPV signal by standard reconstruction and by convolutional modules 1 and 2.

Conventional digital microholography employs numerical reconstruction of holograms to retrieve microscopic object images (*e.g.*, cells, beads), as seen in bright-field microscopy (Figure S3). These require intensive computations for each sample, which often lead to delayed results and need for high-power desktop computing resources (*i.e.*, graphic processing units, GPUs). We previously sought to circumvent such demands on end-users by leveraging cloud computing tactics. While a step forward, the approach did not fully allay cost concerns from cellular data transmission, cloud hardware, and maintenance expenses, notably in resource-limited settings. To realize on-spot detection with cancer screening intentions, we trained deep learning algorithms to differentiate and count the numbers of PS and silica beads (Figure 2A) and their dimers (Figure 2B) directly from diffraction images. These counts were used to determine the assay signal (*S*) and HPV 16 and 18 positivity. Trained algorithms did not require high-power computing resources and were readily installed on-device to achieve independence from internet connections and cloud computing hardware needs.

The architectures for the deep learning analysis were designed to evaluate holograms by two convolutional neural networks (CNNs). For each image, we predicted PS and silica bead counts (Figure 2A) and generated a probability heatmap of dimer positions along with a final dimer count (Figure 2B-C). Positional information was explicitly generated to provide a user-interpretable context for the final assay signal (S). To minimize the computational complexity of this added functionality, our neural network architectures were designed to share as many parameters as possible for the three similar tasks of PS bead, silica bead, and dimer counting. In Module 1, we employ a bifurcated CNN structure to produce PS and silica bead counts. In Module 2, our fully convolutional neural network includes pre-trained parameters used to generate individual PS and silica bead counts to inform dimer count predictions.

During the development and validation, we used the heatmaps to access the accuracy and locations of bead dimers. Using 13,000 images (128 × 128 pixels) for training, our algorithms showed accuracies of 99% for PS beads, 98% for silica beads and 82% for dimers, calculated by a linear correlation between expected and predicted counts on an unseen validation set of sub-images (Figure 2D). On full-sized (2592 × 1944 pixels) holograms, our method also showed a good correlation with the traditional image reconstruction method (*R*^2^ = 0.96, Figure 2E) when generating the final assay signals. Importantly, our deep learning approach was 120-folder faster than the traditional reconstruction method and completed quadruplicate analyses of a full-sized image less than 2 min using a single CPU and 16GB of RAM (Figure 2F).

### *In vitro* characterization

**Figure 3:**
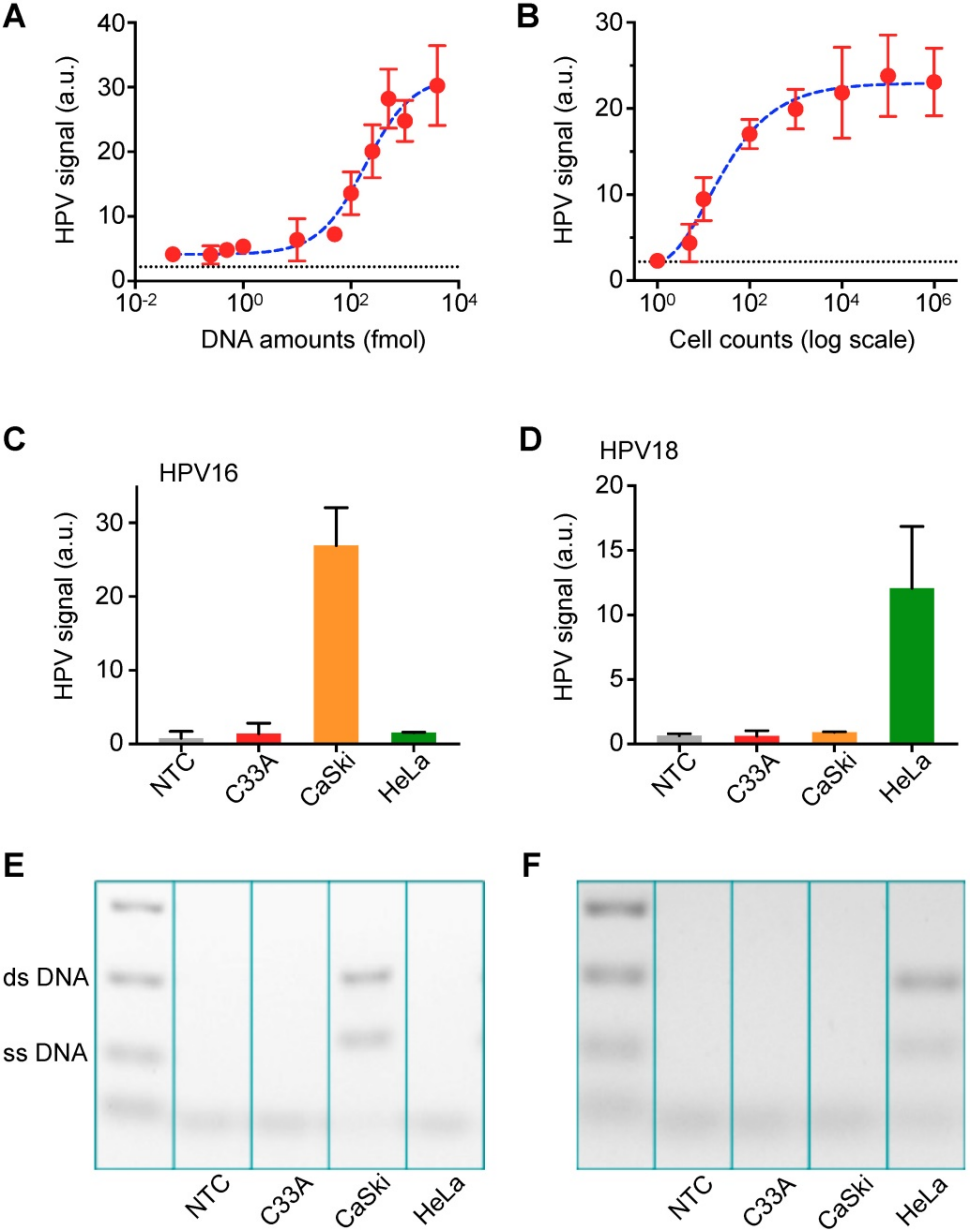
** Titration assay and validation with in vitro cell lines. (A)** The detection sensitivity of the AIM-HPV assay without PCR amplification was determined. Samples containing HPV 16 DNA were serially diluted and detected. The detection limit was ~ 0.09 femtomole. The error bars represent the s.d. of three replicates (*n* = 3). The dashed line presents a cut-off value. **(B)** DNAs extracted from different cell counts were detected. CaSki cell line for HPV 16 DNA was used. The assay demonstrated the detection sensitivity down to a single cell. The error bars represent the s.d. of three replicates (*n* = 3). The dashed line presents a cut-off value. **(C, D)** Three different cancer cell lines (CaSki: HPV16+/18-, HeLa: HPV16-/18+, C33a: HPV16-/18-) were tested with a non-template control (NTC) for HPV 16 (C) and HPV 18 (D). The error bars represent the s.d. of three replicates (*n* = 3). **(E, F)** Gel electrophoresis was used to cross-validate the results for HPV 16 (E) and HPV 18 **(F)** in comparison with AIM-HPV results. The background color is converted for better visualization. The raw gel images are shown in Figure S5.

Next, we determined the sensitivity of the AIM-HPV assay using serially diluted synthetic DNA and HPV-positive cancer cells (CaSki cell line). Without DNA amplification, the assay showed sub-femtomole detection sensitivity (Figure 3A). In terms of cell counts, we were able to detect HPV DNA from a single cell (Figure 3B). To validate the specificity of our assay, we tested three different cell lines (CaSki: HPV16+/18-, HeLa: HPV16-/18+, C33a: HPV16-/18-) for HPV 16 and 18 DNA. Furthermore, we designed DNA probes for β-globin as a control (Figure S4). In the cell line test, we confirmed good specificity for both HPV 16 and 18; DNA from CaSki cell line was only positive for HPV16, and DNA from HeLa cell line was only positive for HPV 18. These are confirmed by both AIM-HPV (Figure 3C-D) and gel electrophoresis (Figure 3E-F, Figure S5). DNA from C33a cell line was negative for both HPV 16 and 18, but positive for β-globin (Figure S4). In the no-template control, no signal was detected for any target sequences.

### Pilot clinical study

**Figure 4:**
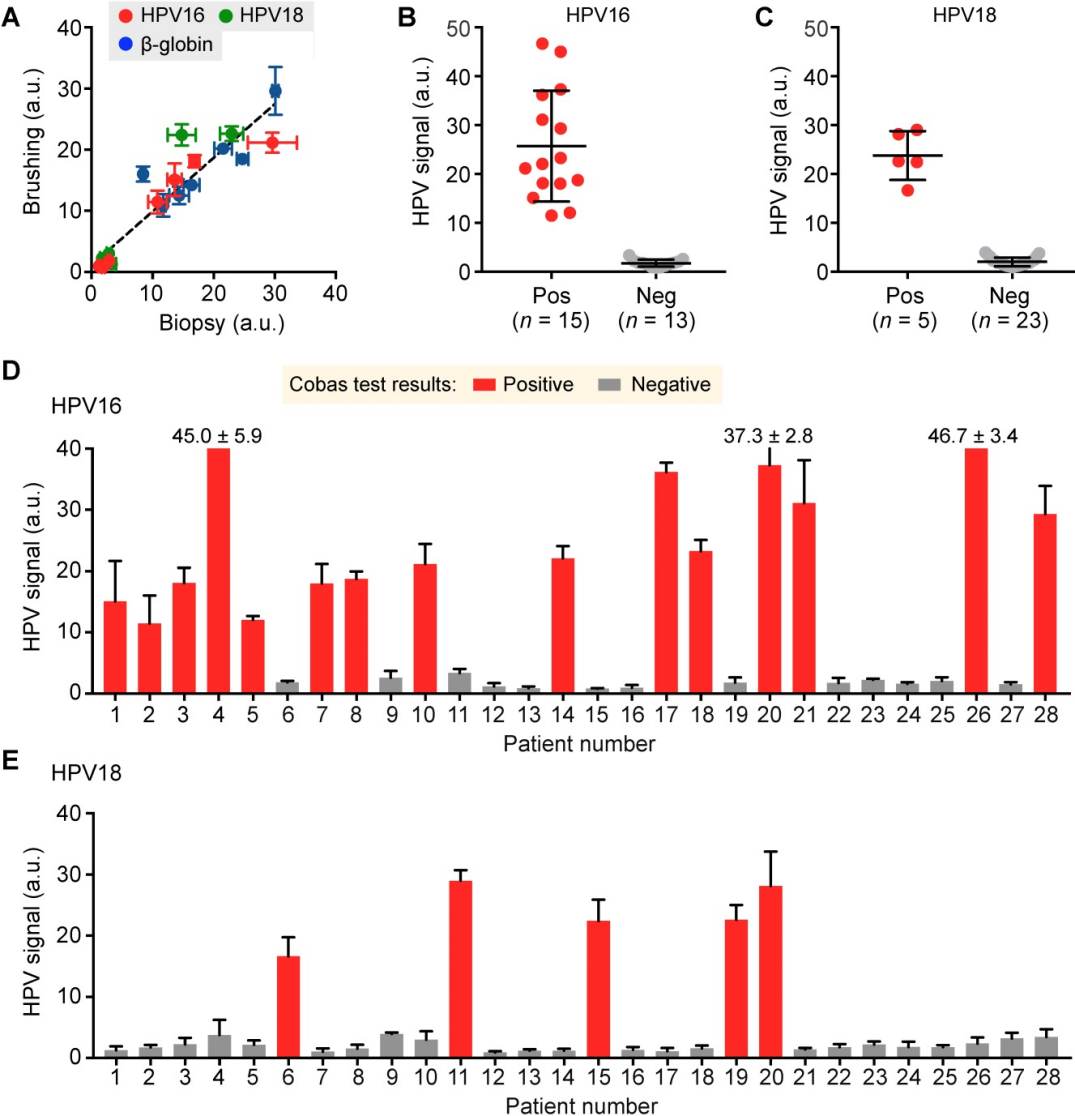
** Detection of HPV 16 and 18 from clinical cervical specimens. (A)** DNAs in cervical specimens collected by brushing and biopsy for HPV 16 (red), HPV 18 (green), and β-globin (blue) were compared. **(B, C)** AIM-HPV assay showed significantly different signals between positive and negative groups for both HPV 16 **(B)** and HPV 18 **(C). (D, E)** Bar graphs of AIM-HPV assay for 28 clinical specimens showed perfect concordance with a gold standard Roche Cobas tests for HPV 16 **(D)** and HPV 18 **(E)**.

Following our preclinical validation, we applied the AIM-HPV assay towards human clinical samples. For 28 patients referred to Massachusetts General Hospital with abnormal pap smear results, we obtained a total of 35 cervical specimens — 28 cervical brushings and seven biopsies. We first compared cervical brushing and biopsy pairs (*n* = 7) of different HPV status (HPV 16+/18-, HPV 16-/18+, and HPV 16-/18-) to test both effectiveness and specimen requirements for the AIM-HPV assay. In all cases, we observed a good correlation of AIM-HPV signals for samples collected by brushings and biopsies (Pearson correlation coefficient *r* = 0.93, *p* < 0.0001; Figure 4A, Figure S6). As a result, we proceeded with cervical brushings (*n* = 28 patients) and screened them for HPV 16 and 18 status. We used a commercially available Cobas HPV test (Roche Diagnostics) clinically used at Massachusetts General Hospital as the gold standard, which reports the positivities for HPV 16 and 18. Our AIM-HPV assay showed significantly different signals (unpaired t-test, *p* < 0.0001) between groups positive and negative for both HPV 16 (25.7 ± 11.4 vs. 1.7 ± 0.7; mean ± s.d., Figure 4B) and HPV 18 (23.8 ± 5.0 vs. 2.0 ± 0.9, Figure 4C). When compared to the Cobas test, the AIM-HPV assay showed full concordance, demonstrating the high accuracy of the assay (Figure 4D-E).

## Discussion

Various studies, including a recently completed large randomized trial of >19,000 women 8, have concluded that HPV testing outperforms the ninety-year old Pap smear test. Notably, HPV testing increased the detection of highest risk pre-cancerous changes (CIN3) and reduced cervical cancer incidence in head-to-head comparisons 8. Moreover, the incidence of cervical adenocarcinoma has increased over the past 20 to 30 years compared to its squamous cell counterpart, which accounts for 90% of all subtypes. Adenocarcinomas, often linked to HPV 18, tend to shed fewer cells and hence challenge cytologic evaluations. These arguments lend further currency to HPV testing. Existing disparities in cervical cancer outcomes domestically and abroad require work that improves screening accessibility through decentralized diagnostics. Success here would increase insight into the racial breadth of high-risk HPV subtypes and inform accurate vaccination strategies.

We advanced AIM-HPV to specifically address the need for decentralized, POC testing performed by lay personnel on easily attained specimens. Here, gentle cervical brushings were employed, and DNA molecules were extracted via disposable syringe filters, key ingredients for feasibility. In the presence of target DNA (HPV16 and 18), PS and silica beads coated with DNA probes complementary to the 3' and 5' ends of HPV target DNA formed dimers. By using two bead types, we discriminated target-specific dimers from self-aggregated beads. Our deep learning algorithms afforded rapid analyses and accurate diagnoses, circumventing costly cloud computing options and reliance on wireless data transmission.

Rapid and accurate algorithmic analyses are key components for translating digital microholography to POC settings. Our use of digital holography provides several advantages over conventional microscopic systems: i) the simple and lens-free optical configuration minimizes our device footprint and cost; ii) we reach field-of-views >100-times larger than conventional microscopes; and iii) both field-of-view and resolution can be quickly improved by upgrading image sensor technology. However, computationally expensive image reconstruction required ether a powerful local server (equipped with graphical processing units) or high-speed internet connection for cloud-based computing, both impractical in low-resource settings. We aimed to address the limitation by adopting deep learning algorithms that can detect target features without computational imaging reconstruction. We previously demonstrated accurate detection of basic cellular holograms and assessed biomarker expression through deep learning algorithms without the need for numeric reconstruction 21,22. To meet the needs of more complex holographic readouts for our DNA analyses, we significantly improve upon binary classification neural networks by providing localization information for multiple or even fractions of microbead dimers, positioned anywhere in an image. The positional information, presented as heat maps, has the following advantages: i) during training, positional information increases the overall object count accuracy; ii) it enables simultaneous, multiplexed detection of different types of objects at different positions (PS, silica and their dimers in this study); and iii) it provides additional information to assess the accuracy of analyses (*i.e.*, secondary analysis to ensure that final dimer counts are accurate). We achieved these multiplexed tasks by sharing parameters and common upstream steps between two modules. In all, this new approach reduces the number of required trainable parameters, the likelihood of overfitting, and computational cost while improving overall detection accuracies.

Expanding on this inaugural DNA microholography platform, we intend to enhance the assay for expanded testing in resource-poor geographies. These will include i) integrating an isothermal amplification method such as recombinase polymerase amplification (Figure S7) or loop-mediated isothermal amplification 23,24; ii) extending the assay for other high-risk HPV strains (*e.g.*, HPV 31, 58) 25,26; and iii) employing reagent lyophilization steps for long-term storage. These new features should further improve detection accuracies and promote rapid adoption for clinical translation testing. Furthermore, rigorous clinical validation in a different environmental setting will be required. Finally, AIM-HPV can be tailored to various other DNA-based biomedical interests through the versatility of its assay. Through continued innovations (*e.g.*, novel microfluidic cartridges^27^), integrated protein and DNA testing could also be achieved with our platform for expanded POC analyses.

## Materials and Methods

### AIM-HPV device

The AIM-HPV device was equipped with a five-megapixel monochromatic complementary metal-oxide-semiconductor (CMOS) image sensor mounted on a USB 2.0 interface board (The Imaging Source). The light source consisted of a 1.4 A high-power 405-nm LED (Thorlabs) heat-sinked by a metal printed circuit board (PCB) and a custom machined aluminum holder, a 220-grit optical diffuser (Thorlabs) and a 50 µm pinhole (Thorlabs). Optical components were aligned by machined acrylonitrile butadiene styrene (ABS) mounts. An integrated 128 × 32 monochrome OLED Display (Wise Semiconductor) provided a real-time view of system status, and a simple momentary switch controlled the LED. Images were directly transferred via USB to a laptop computer. The unit was powered by a regulated 5V, 15W adapter (Meanwell). The device housing was 3D-printed in white photopolymer resin (Formlabs) and was light-proofed using flocking papers (Edmund Optics). The machined-aluminum door was fastened with 1/8 inch neodymium disc magnets (Grainger).

### Cell lines and growth conditions

C33A, CaSki, and HeLa cervical cancer cell lines were purchased from American Type Culture Collection (ATCC). Cell lines were maintained in ATCC recommended growth medium (RPMI-1640 for CaSki; EMEM for C33A and HeLa cells, Cellgro) supplemented with 10% heat-inactivated fetal bovine serum, 100 IU penicillin and 100 µg/mL streptomycin at 37 °C in a humidified atmosphere of 5% CO_2_. All cell lines used for experiments were tested regularly for mycoplasma contamination using a mycoplasma detection kit (MycoAlert™, Lonza). We extracted DNA from approximately 500,000 cells. The total DNA concentration was around 200 ng/µl quantified by Nanodrop. Following that, we used 250 ng DNA for each test.

### Clinical samples processing

The clinical study was approved by the Partners Healthcare Institutional Review Board (Massachusetts General Hospital/Brigham and Women's Hospital). Informed consent was obtained from adult women who were referred to the Colposcopy Clinic for previously abnormal Pap smears. Samples were obtained by brushing and/or cervical biopsy. One clinical provider (M.A.-W.) performed all cervical procedures and provided excess or otherwise discarded ectocervical or endocervical specimens. Biopsies entailed visualizing the exocervix and bathing with 5% acetic acid using clinically standard procedures. Before the use of acetic acid, brushing samples were collected with surgical brushes (Surgipath C-E Brush, Leica Microsystems; Cytobrush Plus GT Gentle Touch, BD Surepath). Cells were carefully removed from the cervical brushes by swirling the brushes in PBS. The entire brushing sample was used up for DNA isolation. DNA was collected fresh within 30 min from the collection of samples; otherwise, the cells were stored at -80 °C. In order to store the samples, we first fixed the samples using BDPhosflow 1x lyse/fix solution and stored them at -80°C until ready to be analyzed. For each patient sample, we have three aliquots for HPV16, 18, and β-globin (internal positive control).

### Fabrication of DNA isolation devices

We used poly(methyl-methacrylate) microbeads (PMMA, 14.7 µm in diameter, Bangs Laboratories) with silica coating^19^ for DNA isolation. The beads (20 mg/mL) were incubated in PBS at pH 8.3, containing 2 mg/mL of dopamine (Sigma Aldrich) for 1 h, after washing with distilled water. The beads were then coated with silica by 1-h incubation in the monosilicic acid solution and washed with distilled water. 50 mg of the silica-coated PMMA beads were dissolved in 500 µL of ethanol, added to a centrifugal filter (Milipore, 0.45 µm pore size), and dried by centrifuge at 9000 g for 30 sec. The packed beads in the filter were further washed with distilled water and ethanol serially by using a centrifuge at 9000 g for 30 sec, then completely dried via centrifugation at 12000 g for one minute. To isolate DNA, cell lysates were prepared and passed through the bead-filled device, followed by washing and elution steps in nuclease-free water.

### HPV DNA detection

Target sequences (~50 nucleotides) unique to HPV 16, HPV 18, and β-globin (control gene) DNA were selected for hybridization-based sandwich assay (Table S1). Pairs of specific oligonucleotide probes (~22 nucleotides) were designed to be complementary to sequences within the target regions of HPV 16, HPV 18, and β-globin DNA with one probe hybridizing to the 5′ end of the target and the other to the 3′ end. One of the probes had a thiol modification at 5' end for attaching it onto the amine-modified polystyrene beads (diameter, 6 µm; Polysciences, 19118-2), and the other had 3' biotin modification to react with streptavidin activated silica beads (diameter, 5 µm; Polysciences, 24755-1). All oligonucleotides used for the probes and target DNA were custom-synthesized by Integrated DNA Technologies. DNA extracted from cell lines and patient samples were amplified by asymmetric PCR. In low resource settings, a portable mini-PCR device (Minipcr, Inc.) could be used for carrying out asymmetric DNA amplification (Figure S8). For asymmetric PCR, forward primers (Table S2) were used in excess of the reverse primer in the ratio of 10:1, 2 µM and 0.2 µM, respectively. We used Maxima Hot Start Taq DNA Polymerase (#EP0601, Thermoscientific), 2 mM magnesium chloride and 0.2 mM dNTPs in the PCR master mix. For hybridization, target DNA sequences were reacted with bead-capture probe conjugates (equivalent to 0.3 pmol capture probe) and incubated in hybridization buffer (DIG Hyb, Roche Diagnostics) with 1% BSA and 100 µg/mL of salmon sperm DNA at 37 °C for 30 min. The unbound target DNA was washed with hybridization buffer using 0.45 µm centrifugal filters (Millipore). The hybridized samples were read using the AIM-HPV device within 1 h (if kept at room temperature) or stored at 4 °C for overnight storage before measurements.

### Convolutional neural network for image analysis

Full-sized sample images were analyzed on a local machine by two convolutional neural networks described below to produce PS bead, silica bead, and PS-silica dimer counts. The second module additionally generated heat maps indicating the expected centroid location of each dimer. These values were then used to determine the AIM-HPV signal and classify positive/negative samples.

**Dataset**. From 12 full-sized sample images, 13,196 sub-images were generated by passing a 128×128 box through each image with a 50% overlap in the x- and y-direction (Figure S9). The dataset was augmented by rotating each sub-image by 90, 180, and 270 degrees. During training, sub-images were also flipped in either the x or y direction with a 50% probability, and diffuse Gaussian noise was added. Finally, sub-images were preprocessed by subtracting the mean pixel value and dividing by the pixel value standard deviation. For final testing, we used 28 samples testing for HPV 16 and 28 samples for HPV 18. Ground truth PS bead, silica bead, and dimer counts were obtained by traditional reconstruction. Corresponding ground truth heat maps were generated by convolving a Gaussian density map with a centroid annotation map.

**Module 1: PS-Si Counting**. This neural network consists of three convolutional layers of increasing depth (Figure 2A), each with a kernel size of 3 and stride 1. Each convolutional layer was followed by a max-pooling layer with kernel size 2 and stride 2. At this point, the model branches into two tasks for PS and Si counting separately. Features extracted from the input image in the final max-pooling layer were duplicated, and a separate set of convolutional kernels were applied to each copy so that PS and Si counting may proceed individually. A global average pooling (GAP) layer was applied to each of the two final convolutional layers to reduce feature dimensionality. Finally, a fully connected (FC) layer was applied to each GAP layer to arrive at final PS and Si counts. All activation functions were leaky rectified linear unit functions with a parameter 𝛼 = 0.1.

This model made use of a regularization method described by Aich et al. in which a class activation map (CAM) is used to improve the final count 28,29. We define *Y* and *y* as our expected and predicted count, respectively, and *H* and *h* as our gaussian annotation and generated CAM layer, respectively. The following optimized loss function was used:


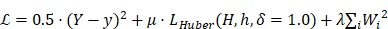


where the first sum term denotes the square count error, the second denotes CAM layer regularization using Huber loss, and the final term is L2 regularization.

**Module 2: Dimer Counting and Localization Module**. The dimer detection network begins with three units of two convolutional and one max-pooling layers (Figure 2B). To transfer the learned capacity of Module 1 to detect PS and Si beads individually, features generated in the final max-pooling layer of Module 1 were concatenated with features generated by the final max-pooling layer of Module 2. Two final convolutional layers were applied after deconvolution, followed by a global sum to arrive at a final count. Thus, the output of the module, *h* is related to the predicted dimer count by 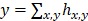. This is also the case for the ground truth count, *Y*, and ground truth heatmap, *H*. The loss function used was:


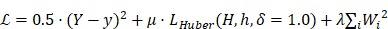


where each sum term is the same as for Module 1.

**Analysis Workflow**. AIM-HPV acquired images were split into sets of smaller images of size 128 pixels by 128 pixels. These images were fed into each of the above modules (Figure S10). Heat maps indicating dimer locations were stitched together into a full-sized image, and total PS, Si, and PS-Si dimer counts were output to generate the final AIM-HPV signal.

## Supplementary Material

Supplementary figures and tables.

## References

[1] Ferlay J, Shin HR, Bray F, Forman D, Mathers C, Parkin DM (2010). Estimates of worldwide burden of cancer in 2008: GLOBOCAN 2008. Int J Cancer.

[2] Adesina A, Chumba D, Nelson AM (2013). Improvement of pathology in sub-Saharan Africa. Lancet Oncol.

[3] Goldie SJ, Gaffikin L, Goldhaber-Fiebert JD (2005). Cost-effectiveness of cervical-cancer screening in five developing countries. N Engl J Med.

[4] Goel A, Gandhi G, Batra S, Bhambhani S, Zutshi V, Sachdeva P (2005). Visual inspection of the cervix with acetic acid for cervical intraepithelial lesions. Int J Gynaecol Obstet.

[5] Hegde D, Shetty H, Shetty PK, Rai S (2011). Diagnostic value of acetic acid comparing with conventional Pap smear in the detection of colposcopic biopsy-proved CIN. J Cancer Res Ther.

[6] Ying H, Jing F, Fanghui Z, Youlin Q, Yali H (2014). High-risk HPV nucleic acid detection kit-the careHPV test -a new detection method for screening. Sci Rep.

[7] Sankaranarayanan R, Nene BM, Shastri SS (2009). HPV screening for cervical cancer in rural India. N Engl J Med.

[8] Ogilvie GS, van Niekerk D, Krajden M (2018). Effect of Screening With Primary Cervical HPV Testing vs Cytology Testing on High-grade Cervical Intraepithelial Neoplasia at 48 Months: The HPV FOCAL Randomized Clinical Trial. JAMA.

[9] Us PSTF, Curry SJ, Krist AH (2018). Screening for Cervical Cancer: US Preventive Services Task Force Recommendation Statement. JAMA.

[10] Malagón T, Kulasingam S, Mayrand MH (2018). Age at last screening and remaining lifetime risk of cervical cancer in older, unvaccinated, HPV-negative women: a modelling study. Lancet Oncol.

[11] Greenbaum A, Zhang Y, Feizi A (2014). Wide-field computational imaging of pathology slides using lens-free on-chip microscopy. Sci Transl Med.

[12] Gurkan UA, Moon S, Geckil H (2011). Miniaturized lensless imaging systems for cell and microorganism visualization in point-of-care testing. Biotechnol J.

[13] Im H, Castro CM, Shao H (2015). Digital diffraction analysis enables low-cost molecular diagnostics on a smartphone. Proc Natl Acad Sci U S A.

[14] Im H, Park YI, Pathania D, Castro CM, Weissleder R, Lee H (2016). Digital diffraction detection of protein markers for avian influenza. Lab Chip.

[15] Xu W, Jericho MH, Meinertzhagen IA, Kreuzer HJ (2001). Digital in-line holography for biological applications. Proc Natl Acad Sci U S A.

[16] Zheng G, Lee SA, Antebi Y, Elowitz MB, Yang C (2011). The ePetri dish, an on-chip cell imaging platform based on subpixel perspective sweeping microscopy (SPSM). Proc Natl Acad Sci U S A.

[17] Greenbaum A, Luo W, Su T-W (2012). Imaging without lenses: achievements and remaining challenges of wide-field on-chip microscopy. Nature Methods.

[18] Pathania D, Im H, Kilcoyne A (2016). Holographic Assessment of Lymphoma Tissue (HALT) for Global Oncology Field Applications. Theranostics.

[19] Hong S, Park KS, Weissleder R, Castro CM, Lee H (2017). Facile silicification of plastic surface for bioassays. Chem Commun.

[20] Chen AA, Gheit T, Franceschi S, Tommasino M, Clifford GM, IARC HPVVSG (2015). Human Papillomavirus 18 Genetic Variation and Cervical Cancer Risk Worldwide. J Virol.

[21] Im H, Pathania D, McFarland PJ (2018). Design and clinical validation of a point-of-care device for the diagnosis of lymphoma via contrast-enhanced microholography and machine learning. Nat Biomed Eng.

[22] Min J, Im H, Allen M, et al.Computational Optics Enables Breast Cancer Profiling in Point-Of-Care Settings ACS Nano. 2018; 12: 9081-90.

[23] Seok Y, Joung HA, Byun JY (2017). A Paper-Based Device for Performing Loop-Mediated Isothermal Amplification with Real-Time Simultaneous Detection of Multiple DNA Targets. Theranostics.

[24] Ding S, Chen R, Chen G (2019). One-step colorimetric genotyping of single nucleotide polymorphism using probe-enhanced loop-mediated isothermal amplification (PE-LAMP). Theranostics.

[25] Muñoz N, Bosch FX, Castellsagué X (2004). Against which human papillomavirus types shall we vaccinate and screen? The international perspective. Int J Cancer.

[26] Yin K, Pandian V, Kadimisetty K (2019). Synergistically enhanced colorimetric molecular detection using smart cup: a case for instrument-free HPV-associated cancer screening. Theranostics.

[27] Turetsky A, Lee K, Song J (2015). On chip analysis of CNS lymphoma in cerebrospinal fluid. Theranostics.

[28] Aich S, Stavness I (1803). Improving object counting with heatmap regulation. ArXiv.

[29] Zhou B, Khosla A, Lapedriza A, Oliva A, Torralba A (2016). Learning Deep Features for Discriminative Localization. Proc IEEE Comput.

