# Supplementary material for: Point-of-care cervical cancer screening using deep learning-based microholography
Source: Theranostics. 2019 Nov 26;9(26):8438–47. doi: 10.7150/thno.37187 (PMC6924258; doi:10.7150/thno.37187)
Supplement: Supplementary file 1 — Supplementary figures and tables. [file thnov09p8438s1.pdf]

Point-of-care cervical cancer screening using deep learning-based microholography

Divya Pathania[1†], Christian Landeros[1,2†], Lucas Rohrer[1,3], Victoria D'Agostino[1,4], Seonki Hong[1], Ismail Degani[1,5], Maria Avila-Wallace[6], Misha Pivovarov[1], Thomas Randall[6], Ralph Weissleder[1,7,8], Hakho Lee[1,8], Hyungsoon Im[1,8*], and Cesar M. Castro[1,9*]

[1] Center for Systems Biology, Massachusetts General Hospital, Boston, MA 02114.
[2] Harvard-MIT Program in Health Sciences and Technology, Massachusetts Institute of Technology, Cambridge, MA 02139.
[3] Department of Health Sciences, Northeastern University, Boston, MA 02115, USA
[4] Department of Bioengineering, Northeastern University, Boston, MA 02115, USA
[5] Department of Electrical Engineering and Computer Science, Massachusetts Institute of Technology, Cambridge, MA 02139, USA
[6] Department of Obstetrics and Gynecology, Massachusetts General Hospital, Boston, MA 02114
[7] Department of Systems Biology, Harvard Medical School, 200 Longwood Ave, Boston, MA 02115
[8] Department of Radiology, Massachusetts General Hospital, Boston, MA 02114
[9] Massachusetts General Hospital Cancer Center, Harvard Medical School, Boston, MA 02114

[†]These authors contributed equally to the manuscript

*Corresponding authors:

Hyungsoon Im, PhD
im.hyungsoon@mgh.harvard.edu
Center for Systems Biology
Massachusetts General Hospital
185 Cambridge St, 5th floor
Boston, MA 02114

Cesar M. Castro, MD
castro.cesar@mgh.harvard.edu
Center for Systems Biology
Massachusetts General Hospital
185 Cambridge St, 5th floor
Boston, MA 02114
(617) 643-3778

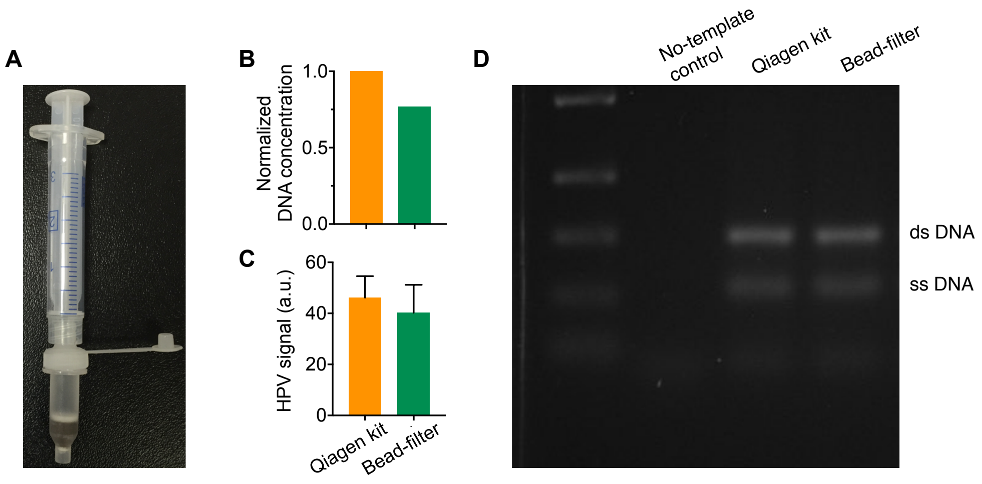

Figure S1. Disposable DNA extraction device. (A) Photography of the DNA extraction device. A disposable filter filled with silica-coated poly(methyl methacrylate) microbeads (PMMA, 14.7 µm in diameter) is connected to a syringe. (B-D) The efficiencies of DNA isolation methods using a commercialized DNA extraction kit (Qiagen kit) and the developed disposable device were compared in terms of total amounts of extracted DNA (B), HPV signal (C), and gel electrophoresis (D) from the same number of cells.

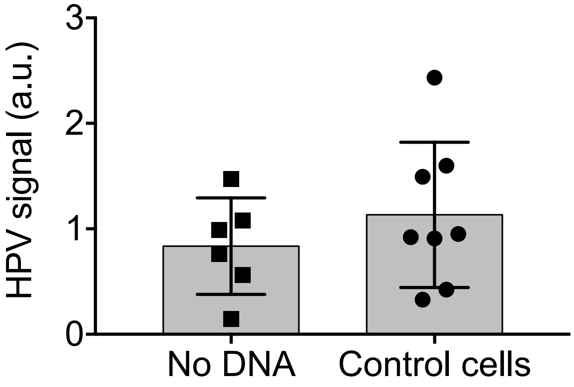

Figure S2. Control experiments. HPV signals were measured with no target DNA (self-aggregation) and control cell lines (C33A and HeLa absence of HPV16 and C33A and CaSki absence of HPV18).

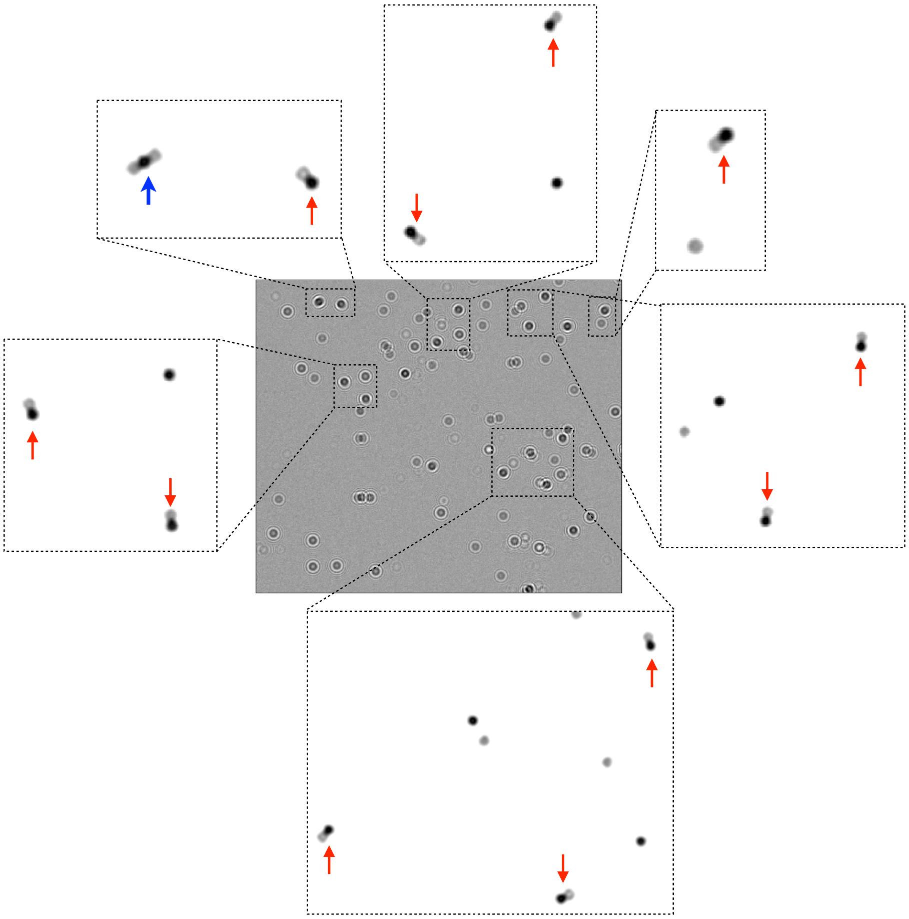

Figure S3. Representative images of dimers. A hologram image is computationally reconstructed to show images of dimers (indicated by arrows) in the zoomed insets. The red arrows indicate single dimers of PS and silica beads. The blue arrow indicates a case of two silica beads binding to a single PS bead, which are counted as two dimers.

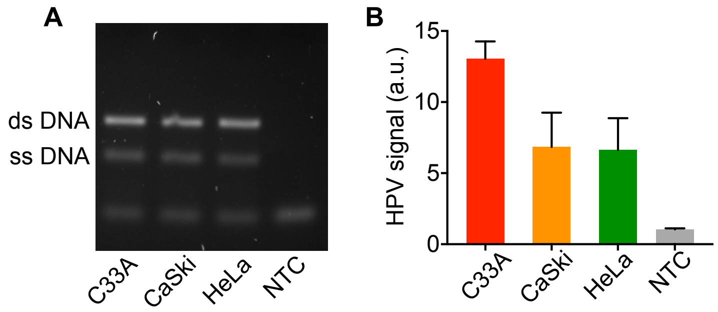

Figure S4. Detection of β-globin DNA as an internal positive control. β-globin DNAs extracted from three different cancer cell lines (CaSki: HPV16+/18-, HeLa: HPV16-/18+, C33a: HPV16-/18-) were detected by (A) gel electrophoresis and (B) AIM-HPV. A non-template control (NTC) was used as a negative control.

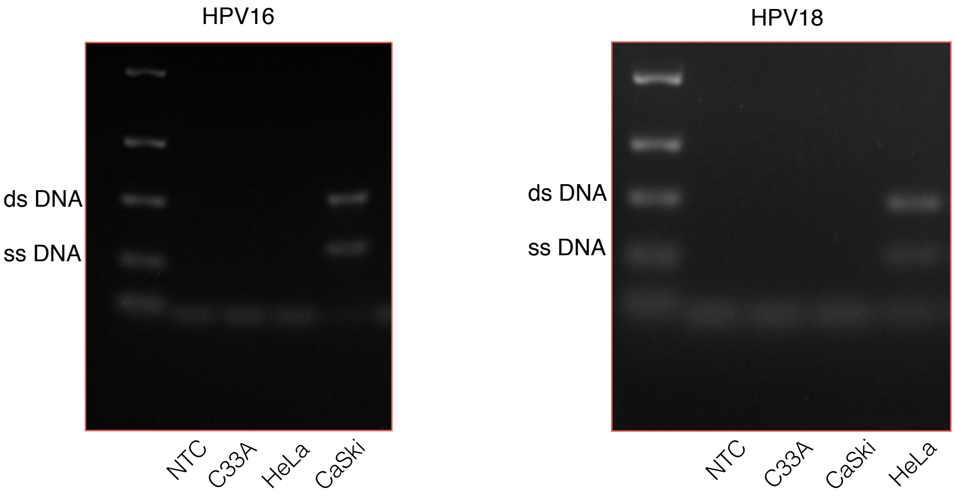

Figure S5. **Unprocessed raw gel data for Figures 3e and f.** The black and white color-scale was reversed, and the order of cell lines was rearranged in Figures 3e and f.

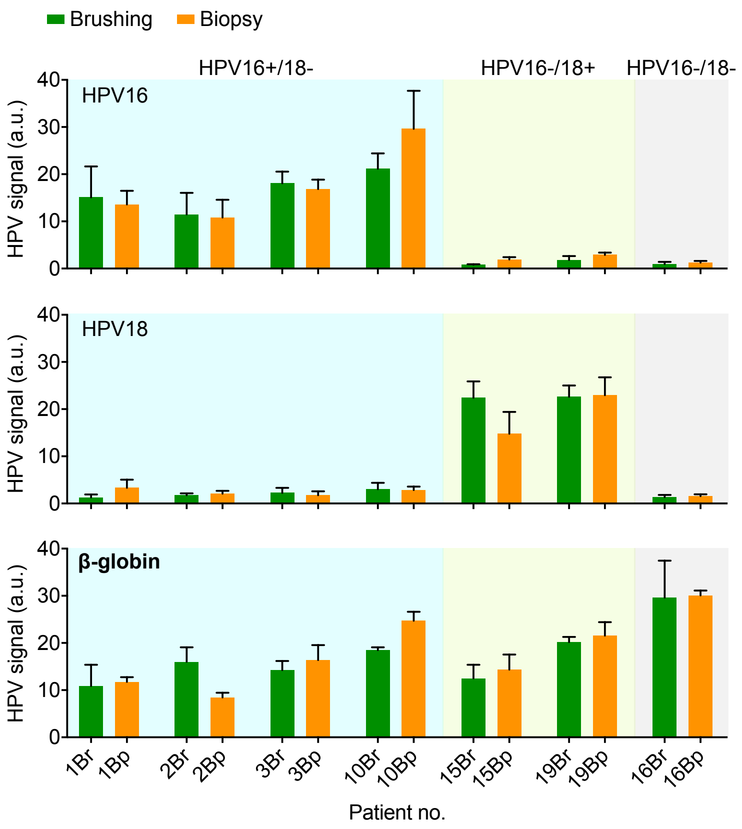

Figure S6. Comparison of brushing and biopsy for cervical specimen collection. For 7 patients, clinical samples were obtained by cervical brushing and biopsy to test both effectiveness and specimen requirements for the AIM-HPV assay.

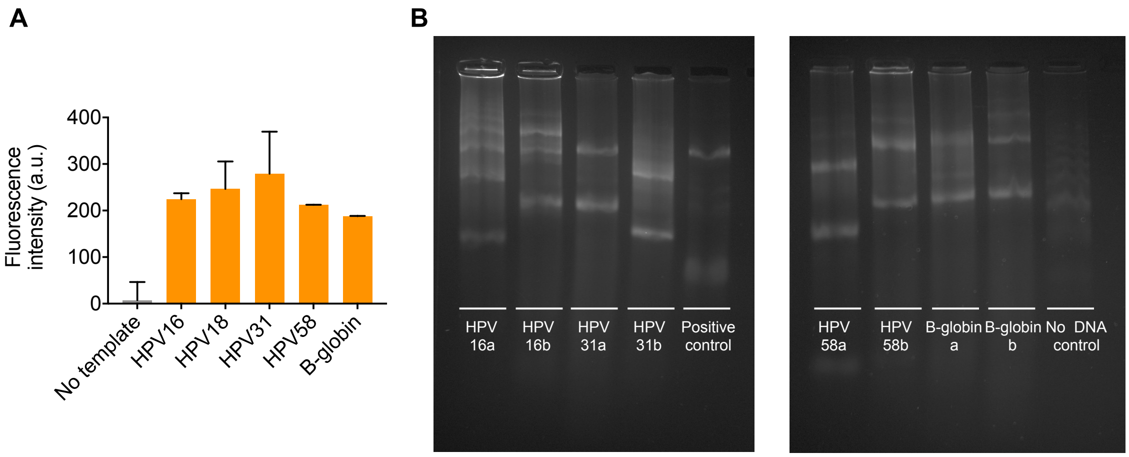

Figure S7 Isothermal amplification for HPV DNA. (A) HPV 16, 18, 31, and 58 DNAs were amplified by a recombinase polymerase amplification (RPA) method at a constant temperature of 37 ℃ and detected by fluorescence measurements. β-globin DNA was used as a positive control, and no template sample was used as a negative control. (B) The amplified DNAs were also detected by gel electrophoresis. Two sets of primers targeting different sequences were tested for each target.

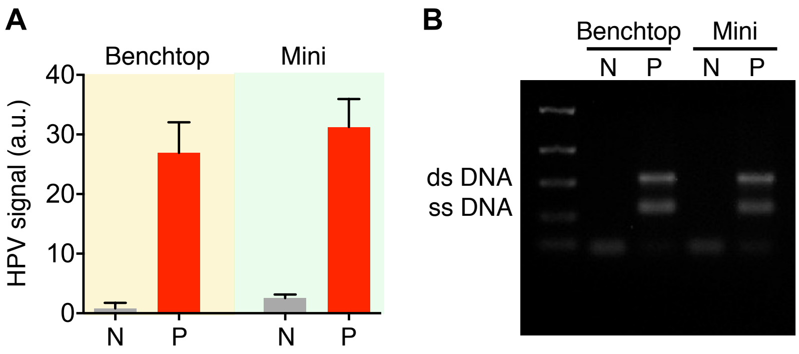

Figure S8. Comparison between conventional benchtop and portable mini-PCR. (A) AIM-HPV and (B) gel-electrolysis were used to detect HPV16 DNA (P). Non-template control (N) was used as a negative control.

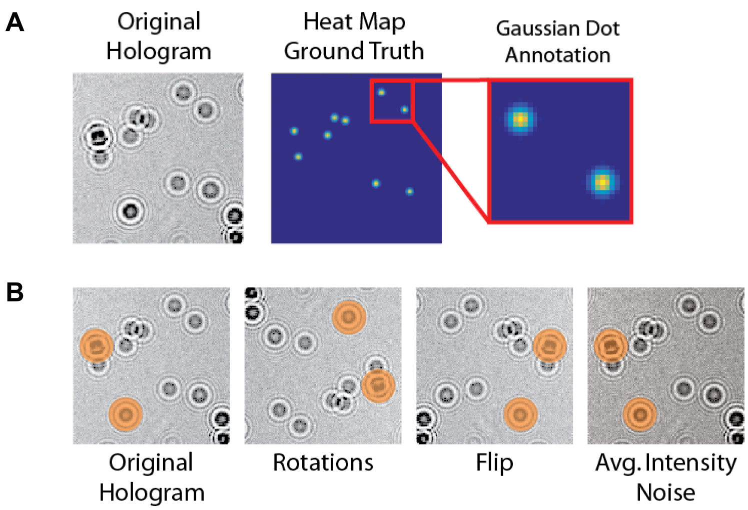

Figure S9. Data Sets for Convolutional Neural Network Training. (A) Ground Truth values are created by summing 2-dimensional gaussian probability maps at each PS, Si, or Dimer centroid detected by computational reconstruction of the hologram images. Centroids in the reconstruction are indicated by x marks. (B) At training time, each input-output pair was either flipped or rotated. Additional model robustness to varying illumination was achieved by introducing background intensity noise, as seen in the right-most panel.

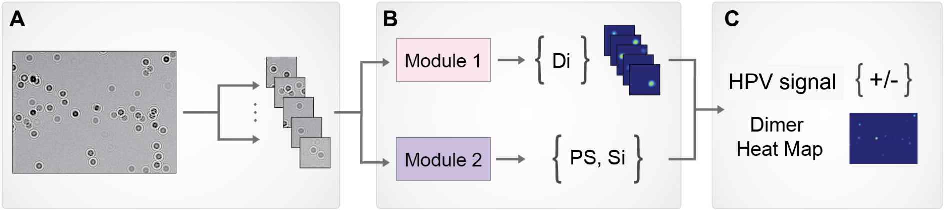

Figure S10. Clinical Sample Analysis Workflow. (A) First, a clinical sample image is split into four sets of 128×128 images. Four sets are designed such that all pixels in the original image are evaluated by deep learning modules at least once. (B) Each set is fed into both the PS-SI counting module (Module 1) and the Dimer localization module (Module 2) to create PS, Si, Dimer counts, as well as heat maps for dimer localizations. (C) PS, Si, and Dimer counts are consolidated to a single HPV signal value (above), which is then used to determine positive/negative evaluations. Heat map pieces are consolidated and averaged to create a final full-sized visualization of probable dimer locations.

Table S1. DNA hybridization probes

Target	Target Sequence	Capture (Thio) Probe	Biotin Probe
**HPV 16**	CTG GTT TGG GCC TGT GTA GGT GTT GAG GTA GGT CGT GGT CAG CCA TTA GGT GT	/5ThioMC6-D/AA AAA AAC ACC TAA TGG CTG ACC ACG	CCT ACA CAG GCC CAA ACC AGA AAA AA/3Bio/
**HPV 18**	GCG CTT TGA GGA TCC AAC ACG GCG ACC CTA CAA GCT ACC TGA TCT GTG CAC GGA ACT	/5ThioMC6-D/AAA AAA AGT TCC GTG CAC AGA TCA GG	CGT GTT GGA TCC TCA AAG CGC AAA AAA/3Bio/
**β-globin**	TGA CAG CCG TAC CTG TCC TTG GCT CTT CTG GCA CTG GCT TAG GAG TTG GA	/5ThioMC6-D/AAA AAA TCC AAC TCC TAA GCC AGT GC	AAG GAC AGG TAC GGC TGT CA AAA AAA/3Bio/

Table S2. DNA PCR primers

Target	Amplicon	Forward Primer	Reverse Primer
**HPV 16**	CTG GTT TGG GCC TGT GTA GGT GTT GAG GTA GGT CGT GGT CAG CCA TTA GGT GT	CTG GTT TGG GCC TGT GTA GGT	ACA CCT AAT GGC TGA CCA CGA C
**HPV 18**	GCG CTT TGA GGA TCC AAC ACG GCG ACC CTA CAA GCT ACC TGA TCT GTG CAC GGA ACT	GCG CTT TGA GGA TCC AAC AC	AGT TCC GTG CAC AGA TCA GG
**β-globin**	TGA CAG CCG TAC CTG TCC TTG GCT CTT CTG GCA CTG GCT TAG GAG TTG GA	TGA CAG CCG TAC CTG TCC TT	TCC AAC TCC TAA GCC AGT GCC
